# Quantification of APOBEC3 Mutation Rates Affecting the *VP1* Gene of BK Polyomavirus In Vivo

**DOI:** 10.3390/v14092077

**Published:** 2022-09-19

**Authors:** Dorian McIlroy, Cécile Peltier, My-Linh Nguyen, Louise Manceau, Lenha Mobuchon, Nicolas Le Baut, Ngoc-Khanh Nguyen, Minh-Chau Tran, The-Cuong Nguyen, Céline Bressollette-Bodin

**Affiliations:** 1 Center for Research in Transplantation and Translational Immunology, UMR 1064, ITUN, Nantes Université, CHU Nantes, INSERM, F-44000 Nantes, France; 2 Faculté des Sciences et des Techniques, Nantes Université, 44093 Nantes, France; 3 Department of Medical Microbiology, Hanoi Medical University, Hanoi 116001, Vietnam; 4 CHU Nantes, Nantes Université, Service de Virologie, 44093 Nantes, France; 5 Molecular Biology and Sequencing Services, CHU Nantes, 44000 Nantes, France; 6 Department of Kidney Diseases and Dialysis, Vietduc University Hospital, Hanoi 110214, Vietnam

**Keywords:** APOBEC, innate immunity, polyomavirus, virus evolution

## Abstract

Mutations in the BK polyomavirus (BKPyV) capsid accumulate in kidney transplant (KTx) recipients with persistent virus replication. They are associated with neutralization escape and appear to arise as a result of cytosine deamination by host cell APOBEC3A/B enzymes. To study the mutagenic processes occurring in patients, we amplified the typing region of the *VP1* gene, sequenced the amplicons to a depth of 5000–10,000×, and identified rare mutations, which were fitted to COSMIC mutational signatures. Background mutations were identified in amplicons from plasmids carrying the BKPyV genome and compared to mutations observed in 148 samples from 23 KTx recipients in France and in Vietnam. Three mutational signatures were consistently observed in urine, serum, and kidney biopsy samples, two of which, SBS2 and SBS13, corresponded to APOBEC3A/B activity. In addition, a third signature with no known etiology, SBS89, was detected both in patient samples, and in cells infected in vitro with BKPyV. Quantitatively, APOBEC3A/B mutation rates in urine samples were strongly correlated with urine viral load, and also appeared to vary between individuals. These results confirm that APOBEC3A/B is a major, but not the only, source of BKPyV genome mutations in patients.

## 1. Introduction

The BK polyomavirus (BKPyV), also known as Human polyomavirus 1, is a small non-enveloped virus with a double-stranded circular genome roughly 5 kb in length. Worldwide, four main genotypes have been identified, of which genotypes I and IV, which are the most prevalent, are divided into subtypes. The four BKPyV genotypes constitute distinct serotypes [1,2] and consequently each genotype is characterized by a specific consensus sequence of the major capsid protein, VP1 [3]. Genotype-specific variation is concentrated in a short stretch of the *VP1* gene, known as the typing region, coding for amino acids 60–84 of the VP1 protein, which comprise the BC-loop on the surface of the BKPyV capsid. The typing region sequence is conserved within subtypes, but it is also the site of mutations that accumulate over time in kidney transplant (KTx) recipients who experience prolonged periods of active BKPyV replication [4]. These mutations allow the virus to escape from the host’s neutralizing antibody response [5,6], and therefore play a role in viral persistence. This is a concern, because chronic polyomavirus associated nephropathy (PyVAN) is associated with subsequent graft loss [7] and there is currently no specific antiviral therapy for PyVAN with proven clinical efficacy.

As a small DNA virus, BKPyV depends on host mechanisms for its genome replication, and presumably has a low intrinsic mutation rate due to the action of host error-correction mechanisms. However, several lines of evidence have indicated that the cytosine deaminase APOBEC3B, and possibly the closely related APOBEC3A, induces mutations in the BKPyV genome. Firstly, BKPyV was shown to induce expression of APOBEC3B, but not other APOBEC3 enzymes in infected renal tubular epithelial cells [8]. These authors also showed that the BKPyV genome is significantly depleted in TCA/TCT motifs, which are consensus sequences for cytosine deamination by APOBEC3A and APOBEC3B, implying that the virus has evolved to become less susceptible to editing by these enzymes. Secondly, Peretti et al. used next-generation sequencing methods to analyze intra-patient BKPyV sequence polymorphism in samples from two patients with PyVAN caused by gIV BKPyV infection [6]. They noted that the VP1 mutations observed in their samples occurred at APOBEC3A/B target sites. They also showed that renal tubular epithelial cells in graft biopsies showed nuclear expression of APOBEC3 proteins. As the antibody used for staining cross-reacted with APOBEC3A, 3B, and 3G, it was not possible for the authors to formally determine which of these three enzymes was present. Finally, it has recently been reported that BKPyV infection induces expression of both APOBEC3A and APOBEC3B in primary urothelial cells [9]. This appears to be the mechanism underlying the epidemiological link between previous BKPyV infection and bladder carcinoma [10], because bladder cancer genomes carry a strong APOBEC3 mutagenesis signal [11,12,13].

The recognition that different cancers have distinct patterns of somatic mutations, related to the specific mutagenic processes that induce these cancers, has led to the definition of a series of single base substitution (SBS) and double base substitution (DBS) signatures [14,15]. Many, though not all, SBS signatures have been related to specific mutagenic processes, and in particular, SBS2 and SBS13 correspond to DNA editing by APOBEC family cytidine deaminases. A number of bioinformatic tools have been developed to extract SBS profiles from sequencing data, and in the present work, we adapt one of these tools, the R/Bioconductor Mutational Patterns package [16,17], to analyze mutations in the typing region of the *VP1* gene amplified from a cohort of patients in France and Vietnam. In previous work [5] we had used the breseq package [18] to follow the accumulation of VP1 mutations over time in KTx recipients with persistent BKPyV replication, and inspection of this data showed that many mutations, present at frequencies <1%, had been excluded from the analysis. We therefore speculated that further information could be derived from these low frequency mutations in the VP1 typing region and developed a bioinformatic pipeline to study them. Our overall aims were firstly, to determine whether APOBEC3A/B editing of BKPyV genomes can be identified in next-generation sequencing data; secondly, to compare the extent of APOBEC3A/B editing observed in samples from different KTx recipients; and finally, to identify additional sources of BKPyV mutations in vivo.

## 2. Materials and Methods

### 2.1. Patients

Patients in the present study were included retrospectively in Nantes and in Hanoi, based on the level of viral load recorded in the hospital laboratory databases. Patients in Nantes were transplanted between 2017 and 2019 and had previously been included in a prospective observational study, approved by the local ethics committee and declared to the French Commission Nationale de l’Informatique et des Libertés (CNIL, n°1600141). Patients in Hanoi were transplanted between 2014 and 2017. All patients in Nantes and Hanoi gave informed consent authorizing the use of archived urine (Hanoi and Nantes), blood (Nantes), and biopsy (Nantes) samples for research protocols. Anonymized clinical and biological data for these patients were extracted from the hospital databases. Of the 15 Nantes patients, 9 had biopsy confirmed PyVAN, 3 had suspected/presumed PyVAN, and 3 had no PyVAN. Among the 8 Hanoi patients, 2 had biopsy confirmed PyVAN, and 6 had no PyVAN. 

### 2.2. Amplification and Sequencing of VP1 Typing Region from Patient Samples

The typing region was amplified from urine and EDTA whole-blood extracted DNA (QiaSymphony, Qiagen, Hilden, Germany) using primers originally described in [19] extended with eight different, unique and base-balanced 8 bp barcodes added to the 5′ end of each forward and reverse primer. This provided a 16 primer set from which each forward and reverse primer were used in combination to create a dual barcode sample index (Table 1). Each pool of 8 samples included either 2 or 3 control samples composed of a mix of plasmids coding for with either the wild-type BK MM genome sequence, or the BK-MM genome incorporating the BC-loop mutations A72V, E73Q, E82Q, or the mutations A72V, E73K. For these controls, plasmids were first mixed in known proportions, cut with BamHI, then religated, purified, and re-quantified. 1 pg of each plasmid mix (~10^5^ copies) was used as a template in the PCR reaction. 

Barcoded VP1 sequences were amplified in a reaction volume of 25 µL containing 5 µL extracted viral DNA, 0.5 U Herculase II (Agilent, Santa Clara, CA, USA), 250 µM each dNTP, 0.25 µM primers, and 1× PCR buffer. After initial denaturation at 95 °C for 5 min, amplification was performed for 35 cycles on a Bioer LifePro Thermal Cycler (Grosseron, Coueron, France). Cycling conditions were 95 °C for 30 s, 54 °C for 30 s, and 72 °C for 30 s, with final extension at 72 °C for 7 min. PCR products were purified using columns (NucleoSpin Gel and PCR Clean-up, Macherey-Nagel, Düren, Germany) and eluted into 5 mM Tris/HCl, pH 8.5 buffer. Amplicon lengths and concentrations were measured with a LabChip GX (Caliper Life Sciences, Waltham, MA, USA) using a high sensitivity 1K chip. PCR products were then pooled in equimolar proportions, and libraries were prepared using NEBNext Ultra II DNA Library Prep Kit for Illumina (New England Biolabs, Ipswich, MA, USA) and tagged with barcoded i5 and i7 primers for Illumina 2×250 bp sequencing on a MiSeq instrument (v2 Nano Reagent Kit) at the CHU Nantes Molecular Biology and Sequencing platform.

### 2.3. Bioinformatic Analysis

Raw Illumina paired-end reads were quality trimmed, checked for N content, and length filtered using Cutadapt version 3.5 [20]. Reads were then 5′ trimmed and assigned to each patient’s sample using Flexbar version 3.5.0 [21]. To detect and annotate mutations rare mutations present at frequencies down to 0.05%, reads were first mapped onto the reference genomes for each BKPyV genotype (Table S2) with minimap2 version 2.24 [22], with sequences numbered from the start of the *Agno* gene, then Samtools version 1.7 [23] was used to generate each patient’s consensus sequence from the first available clinical sample. This consensus sequence was then used as a reference by LoFreq version 2.1.5 [24] to identify mutations in reads represented in the sorted .bam file, which were output in .vcf format. Since the plasmid controls contained mixes of wild-type and mutant sequences at three positions in the amplicon, mutations at these positions were removed from control sample .vcf files before downstream analysis using vcftools version 0.1.16 [25]. Since BKPyV-specific PCR amplicons were analyzed, no steps were taken to exclude human genomic sequences. 

In order to apply the Mutational Patterns package to the .vcf files produced by LoFreq, it was necessary to prepare a reference genome in BSGenome format [26]. This was done using the BKPyV reference genome sequences (Table S2) to forge a BSGenome data package using the circ-seqs field to specify that BKPyV genomes are circular. In this data package, each BKPyV reference genome is represented as a distinct chromosome, so that the same BSGenome object can be used for all BKPyV genotypes.

In Mutational Patterns, each observed mutation was counted as a single event, regardless of its frequency in the viral population, and the trinucleotide sequence context was plotted using the plot_96_profile function. Mutations were fitted to SBS signatures using fit_to_signatures_strict, with the max_delta parameter set to 0.01. To determine the average contribution of each SBS signature, fitting was performed with 50 bootstrap iterations, then visualized either as a dotplot or a bubble plot. The mean and standard deviation of the bootstrap iterations were exported using the summarize function in the dplyr package. Mutational Patterns and other R packages were run in R 4.1.0 in RStudio.

### 2.4. Cell Culture Model

T-REx-293 cells expressing doxycycline inducible APOBEC3B (A3Bi) and APOBEC3B with an inactivated catalytic domain (DCM) [27] were kindly provided by Dr R.S. Harris (University of Minnesota, USA). They were cultured in DMEM medium (Dutscher) supplemented with 10% complemented FCS, 2 mM Glutamine, 100 U/mL penicillin, 100 µg/mL streptomycin, and 200 µg/mL hygromycin at 37 °C in 5% CO_2_. 5 × 10^4^ cells were infected with a BK-MM virus at MOI of 1 FFU/cell, then doxycycline was added at 1 mM or 25 mM doxycycline at 24 hpi. Medium was changed every two days to maintain the doxycycline concentration, then cells were harvested at 11 dpi. Cells were lysed by resuspension in 100 µL hypotonic lysis buffer consisting of 25 mM Sodium Citrate pH 6.0, 1 mM CaCl_2_, 1 mM MgCl_2_ and 5 mM KCl. Cells were sonicated in a Bioruptor Plus device (Diagenode) for 10 min at 4 °C with 5 cycles of 1 min. ON/1 min OFF. Type V neuraminidase (Sigma, Saint-Quentin-Fallavier, France) was added to a final concentration of 1 U/mL and the extract was incubated for 30 min. at 37 °C. The pH was neutralized by adding 100 µL of 1 M HEPES buffer pH 7.4, then 1 µL (25 U) Benzonase (Sigma) was added, followed by 2 h incubation at 37 °C. Virus present in this extract was quantified by qPCR as described in [28], after releasing encapsidated DNA by proteinase K digestion (200 µg/mL proteinase K for 1 h at 55 °C, followed by 10 min at 95 °C). This process was repeated for four rounds of virus replication, infecting 1 × 10^5^ A3Bi or DCM cells at 10^4^ BKPyV genome copies per cell, before the addition of doxycycline. Finally, the round 4 proteinase K extract was diluted 80×, and 5 µL (corresponding to 2.5 × 10^6^–2.5 × 10^7^ genome copies) was used for high fidelity PCR using 1U Q5 polymerase (NEB), 0.2 µM of primers listed in Table S1, and 1× Q5 reaction buffer in a 25 µL reaction volume. PCR cycling conditions were 98 °C for 1 min. Followed by 30 cycles at 98 °C 20 s/56 °C 20 s/72 °C 20 s and a final extension time of 2 min. PCR products were purified using columns (NucleoSpin Gel and PCR Clean-up, Macherey-Nagel) and eluted into 5 mM Tris/HCl, pH 8.5 buffer, then sent for Illumina amplicon sequencing, without sample pooling, to Genewiz (Leipzig, Germany). FastQ files were downloaded, then processed as described above.

## 3. Results

### 3.1. APOBEC3A/B Signatures Can Be Detected in Urine, Plasma, and Kidney Biopsy Samples

To test whether rare APOBEC3A/B induced mutations could be detected by deep sequencing, the VP1 typing region was amplified from 58 clinical samples from 7 KTx recipients (11 graft biopsies, 16 plasma, and 31 urine) and 22 plasmid controls. Individual samples were amplified by dual barcoded primers, then pooled in batches of 8 amplicons, in which each pool contained at least two PCR products amplified from a plasmid carrying the full genome of the reference BK-MM strain. After Illumina sequencing, primer barcodes were used to demultiplex the samples in each pool, reads were aligned against the consensus sequence from the first available sample from each patient, then the LoFreq package was used to identify rare mutations, present at frequencies as low as 0.05% of reads. The profiles of these rare mutations were then analyzed in Mutational Patterns. Firstly, the mutations listed in the .vcf files were grouped by sample type in order to determine whether PCR products amplified from clinical samples contained specific types of mutations distinct from those observed in plasmid controls. 

**Figure 1:**
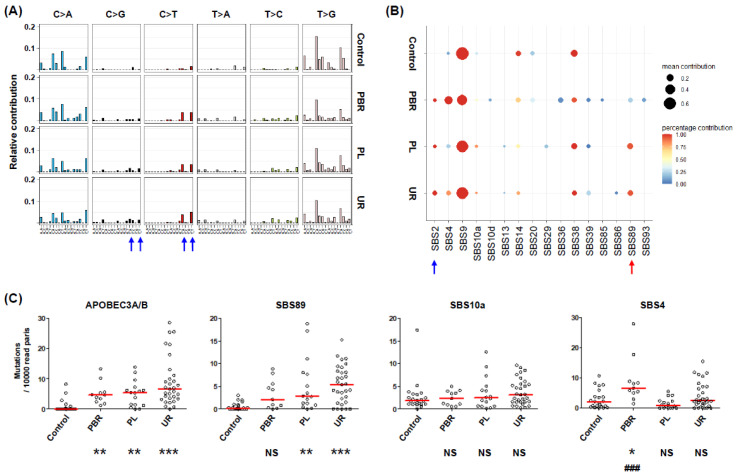
Identification of mutational profiles in PCR products from clinical samples. (**A**) Type and context of rare mutations present in VP1 typing region PCR products amplified from plasmid controls (Control), kidney biopsies (PBR), plasma (PL), and urine (UR) samples from KTx recipients with BKPyV replication. Blue arrows—C>G and C>T mutations in the context of TCA /TCT trinucleotides. (**B**) Fitting of observed mutations to SBS signatures over 50 bootstrap iterations. Arrows—SBS signatures consistently present in clinical samples but were absent from controls. (**C**) Data are presented as the number of each type of mutation per 10,000 read pairs in PCR products amplified from plasmid controls (Control), kidney biopsies (PBR), plasma (PL), and urine (UR) samples from KTx recipients with BKPyV replication. Each symbol represents the mean of 50 SBS fitting iterations of data from a single sample, and red lines indicate the median for each group. Sample types were compared by the Kruskal–Wallis test, and Dunn’s multiple comparison post-hoc test. Symbols indicate: * *p* < 0.05, ** *p* < 0.01, *** *p* < 0.001, NS: Not Significant, compared to plasmid controls. ### *p* < 0.001 compared to PL group. All other differences between PBR, PL, and UR groups were not significant.

Mutations in control samples were predominantly C->A and T>G transversions (Figure 1A), presumably due to errors induced by the Herculase II DNA Polymerase used in the PCR reaction. PCR products from patient samples, but not from plasmid controls, carried additional mutations, including C>G transversions and C>T and T>C transitions. Peaks (blue arrows, Figure 1A) could be seen for C>G and C>T mutations in the context of the TCA and TCT trinucleotides—the optimum site for APOBEC3A/B cytosine deamination. Fitting the observed mutations to Cosmic SBS signatures resulted in the detection of strong signals for SBS9, SBS14, and SBS38, and a less consistent detection of SBS4, SBS10a, and SBS20 in all sample types—including the plasmid controls (Figure 1B). These signatures are therefore likely to represent the polymerase errors shown in Figure 1A. However, SBS2 (blue arrow, Figure 1B) and, to a lesser extent, SBS13, corresponding to APOBEC3 editing, and an additional signature, SBS89 (Red arrow, Figure 1B), were found only in clinical samples, and not in PCR products amplified from plasmid controls.

Fitting mutations to SBS signatures is a probabilistic process, so to quantify the number of mutations for specific SBS signatures in each sample, we used the mean calculated from 50 bootstrap iterations, then normalized this to the number of read pairs per sample (Figure 1C). The number of SBS2 and SBS13 mutations were summed in order to calculate the total number of APOBEC3A/B mutations. Significantly more APOBEC3A/B mutations per 10^4^ read pairs were found in amplicons from clinical samples compared to plasmid controls. and there was no significant difference in the rate of APOBEC3A/B mutations detected in DNA extracts from kidney biopsies, plasma, or urine samples. Three other mutational signatures which appeared to be more readily detected in clinical samples than in plasmid controls (Figure 1B), SBS89, SBS10a, and SBS4, were also analyzed in this way. Compared to plasmid controls, the number of SBS89 mutations per 104 read pairs was significantly higher in plasma and urine samples, and although the same tendency was seen for kidney biopsies, it did not reach statistical significance (Figure 1C), possibly due to the low number of biopsy samples analyzed. In contrast, the rate of SBS10a mutations was equivalent in controls and clinical samples, indicating that it was a non-specific background signal. The SBS4 mutation rate showed a more complex pattern, since it was significantly higher in kidney biopsies compared to plasmid controls and, surprisingly, plasma samples (Figure 1C). 

Statistical analysis therefore confirmed that APOBEC3A/B and SBS89 were the only two mutational patterns that were specifically and consistently detected in PCR products from clinical samples, and we then concentrated on these two mutational signatures.

### 3.2. APOBEC3A/B Mutation Rate Varies between Patients, Whereas SBS89 Mutation Rate Varies between BKPyV Genotypes 

**Figure 2:**
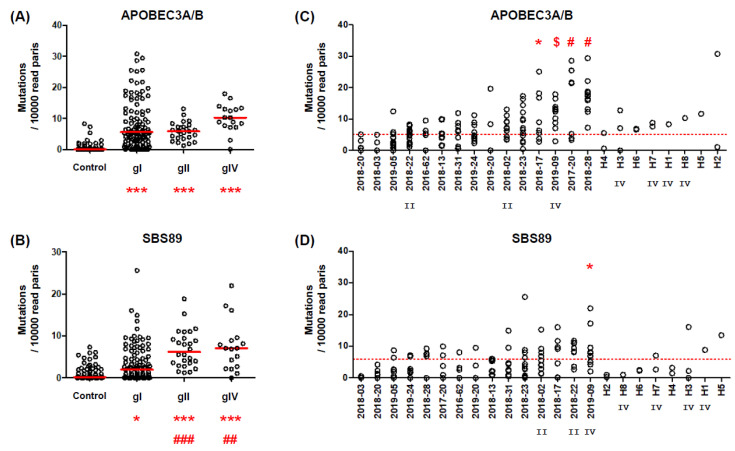
APOBEC3A/B and SBS89 mutation rates in relation to virus genotype and patient. (**A**) APOBEC3A/B and (**B**) SBS89 mutation rate by virus genotype. Each symbol represents the mean of 50 SBS fitting iterations of data from a single sample, and red lines indicate the median for each group. Sample types were compared by the Kruskal–Wallis test, and Dunn’s multiple comparison post-hoc test. Symbols indicate: * *p* < 0.05, *** *p* < 0.001, compared to plasmid controls. ## *p* < 0.01, ### *p* < 0.001 compared to gI virus. All other differences between groups were not significant. (**C**) APOBEC3A/B and (**D**) SBS89 mutation rate by patient, arranged in order of lowest to highest mean among Nantes and Hanoi patients. Each symbol represents the mean of 50 SBS fitting iterations of data from a single sample. APOBEC3A/B mutation rates among Nantes patients were compared by one-way ANOVA, and Dunnett’s post-hoc test, with patient 2018-20 as the comparator. Symbols indicate: * *p* < 0.05, $ *p* < 0.01, # *p* < 0.001. No other significant differences were observed. Dotted red lines show the 95th percentiles of APOBEC3A/B and SBS89 mutation rates observed in plasmid controls (n = 60). BKPyV genotypes II and IV are indicated beneath the patient code.

Having confirmed that APOBEC3A/B and SBS89 mutations in the BKPyV typing region could be detected in PCR products amplified from patient urine, as well as plasma and kidney biopsies, we then analyzed urine samples from a further 8 patients in Nantes (total of n = 15 KTx recipients, median = 9 samples per patient), 8 patients in Hanoi (median = 2 samples per patient), and 38 additional plasmid controls. The full data set allowed us to investigate the association between virus genotype and mutation rates, and to explore inter-patient variability in APOBEC3A/B and SBS89 mutation rates. The APOBEC3A/B mutation rate did not significantly differ between BKPyV genotypes I, II, and IV (Figure 2A), whereas median SBS89 mutation rates were significantly higher in gII (6.2 mutations/10^4^ read pairs) and gIV (7.0 mutations/10^4^ read pairs) viruses compared to genotype I (1.9 mutations/10^4^ read pairs) viruses (Figure 2B).

Interpatient variability was apparent in the APOBEC3A/B mutation rate, with the mean number of APOBEC3A/B mutations per 10^4^ read pairs varying from 2.0 to 18.8, with statistically significant differences between patients (Figure 2C) by one-way ANOVA, using the patient with the lowest mean APOBEC3A/B mutation rate (i.e., patient 2018-20) as the comparator. In contrast, the SBS89 mutation rate showed lower variability, ranging from 0.2 to 8.2 (Figure 2D), with only patient 2019-09, infected by a gIV virus, showing a significantly higher SBS89 mutation rate than either patient 2018-03 (n = 3 samples) or patient 2018-20 (n = 5 samples) as the comparator. The values observed in patients from Hanoi were in the same range as those found in patients from Nantes, although the association between gIV BKPyV and higher SBS89 mutation rates was not recapitulated in this limited sample (Figure 2D). 

**Figure 3:**
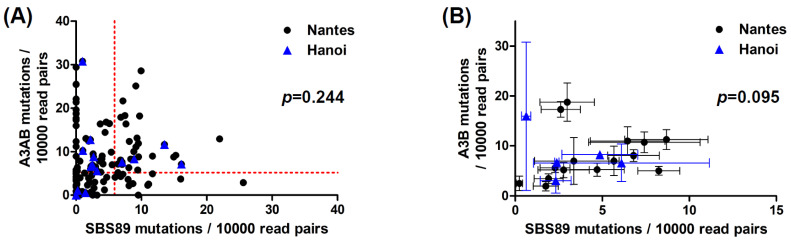
Association between APOBEC3A/B and SBS89 mutation rates. (**A**) Correlation between APOBEC3A/B and SBS89 mutation rates in individual samples. Dotted red lines show the 95th percentiles of APOBEC3A/B and SBS89 mutation rates observed in plasmid controls (n = 135). (**B**) Correlation between mean APOBEC3A/B and SBS89 mutation rates in 15 Nantes patients and the 5 Hanoi patients for whom at least two data points were available. Error bars show SEM. The Spearman’s rank correlation coefficient was used for statistical analysis of the complete data set without distinction between Nantes and Hanoi patients.

Within individual samples, the rates of APOBEC3A/B and SBS89 mutation were not correlated (Figure 3A), however, there was a weak tendency for the mean and SBS89 mutation rates to be correlated within patients (Figure 3B). That is, patients with a high mean APOBEC3A/B mutation rate tended to have a higher SBS89 mutation rate (*p* = 0.095). Interestingly, the three patients with the highest mean APOBEC3A/B mutation rate stood out from this trend, and if data from these three patients were excluded, then the association between APOBEC3A/B and SBS89 mutation rates became highly significant (*p* < 0.001).

### 3.3. Longitudinal Variation in APOBEC3A/B and SBS89 Mutation Rates in Patients 

**Figure 4:**
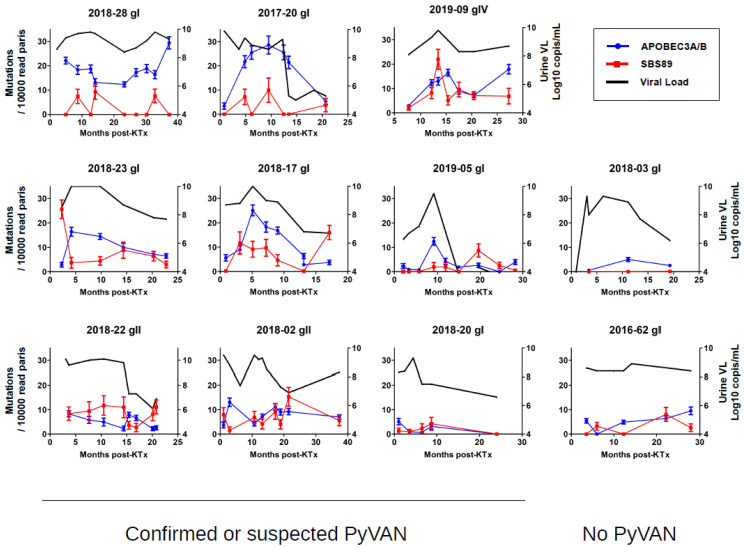
Intrapatient variation in APOBEC3A/B and SBS89 mutation rates over time.

Although interpatient differences were observed in APOBEC3A/B mutation rates, there was also extensive variability of both APOBEC3A/B and SBS89 mutation rates between different samples from the same patient (Figure 2C,D). In order to investigate intrapatient variability in APOBEC3A/B and SBS89 mutation rates, we plotted these parameters and urine viral load over time post-KTx in patients with confirmed or suspected PyVAN (Figure 4). Patient 2018-28 had a uniformly high (i.e., >10) APOBEC3A/B mutation rate, together with consistently high viruria, while in patients 2017-20, 2019-09, 2018-23, 2018-17, and 2019-05, the APOBEC3A/B mutation rate tracked changes in the viral load. In two cases (2017-20 and 2019-09), the peak APOBEC3A/B mutation rate was delayed compared to peak viral load. Finally, three patients (2018-22, 2018-02, and 2018-20) showed consistently low APOBEC3A/B mutation rates, despite viruria >8 log10 copies/mL, which was similar to what was observed in two patients without PyVAN. Overall, APOBEC3A/B mutation rates in urine samples were strongly correlated with urine viral load (Spearman r = 0.52, *p* < 0.001). In contrast, the SBS89 mutation rate was not significantly correlated with viral load (Spearman r = 0.19, *p* = 0.07), although in some individuals with gII or gIV BKPyV, for example, patients 2019-09 and 2018-22, SBS89 mutation rates appeared to coincide with viruria.

Viral load (Log10 copies/mL, right axis, Black line), APOBEC3A/B (blue), and SBS89 (red) mutation rates (mutations per 10,000 read pairs, left axis) in sequential urine samples are plotted against time post KTx. Error bars show the 95% confidence interval of the mean APOBEC3A/B and SBS89 mutation rates in the sample calculated from 50 SBS fitting iterations.

### 3.4. SBS89 Mutational Profile Is Found during BKPyV Replication in Cell Culture

**Figure 5:**
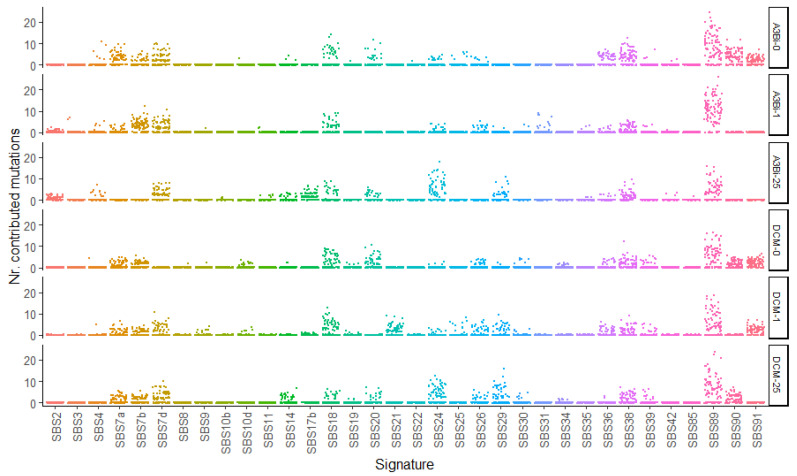
Mutational patterns detected in BK-MM strain BKPyV replicating in cell culture.

To recapitulate our observations in clinical samples, we analyzed the low frequency mutations present in MM strain BKPyV after four rounds of replication in HEK-293 derived cells expressing doxycycline inducible APOBEC3B (A3Bi) and APOBEC3B with an inactivated catalytic domain (DCM) [27]. Six parallel cultures were set up, consisting of infected A3Bi cells and DCM cells with 0, 1, and 25 ng/mL doxycycline. The typing region of VP1 was amplified, sent for Illumina sequencing, then the low-frequency mutations present in the amplicons were analyzed as above. In all six cultures, the strongest mutational pattern identified was SBS89 (Figure 5), and a weak SBS2 signal, corresponding to APOBEC3A/B activity, was only detected in the virus that had replicated in A3Bi cells in the presence of doxycycline. Due to the high fidelity of the Q5 polymerase, the SBS9 and SBS14 signals that were seen in the clinical samples amplified with Herculase II DNA Polymerase were not observed in this experiment. We therefore conclude that the SBS89 signal represents mutations that accrue during virus replication.

Four rounds of BKPyV replication were performed on HEK-293 derived cells expressing doxycycline inducible APOBEC3B (A3Bi) and APOBEC3B with an inactivated catalytic domain (DCM) in the absence (A3B-0, DCM-0) or presence of doxycycline (1 ng/mL A3B-1, DCM-1; 25 ng/mL A3B-25, DCM-25). Observed mutations were fitted to SBS signatures over 100 bootstrap iterations. Results are presented as a plot where each point represents the results of one fitting iteration.

## 4. Discussion

The seminal studies of APOBEC enzyme editing of DNA virus genomes used 3D-PCR to specifically amplify hypermutated viral genomes [29,30], and more recently, several reports have used in-house scripts to detect APOBEC mutations in HPV genomes at different stages of HPV infection [31,32,33,34]. In this study we used a different approach to detect and quantify APOBEC3A/B editing of BKPyV genomes, based on the fitting of mutations observed in NGS data to previously described SBS signatures. Compared to 3D-PCR, our approach may be less sensitive, but unlike 3D-PCR, it does allow quantitative comparisons between samples, by counting the number of observed APOBEC3A/B mutations per 10^4^ read pairs. Using a script to specifically count mutations occurring at consensus APOBEC3A/B sites could achieve this more directly and with greater precision, but such targeted approaches inevitably ignore other types of mutations potentially present in the data. Therefore, while the experimental and bioinformatic methodology that we used may not be the most sensitive or precise technique to detect APOBEC3A/B editing of BKPyV genomes in clinical samples, it did have the potential to identify and quantify additional mutational signals.

We found that the signature fitting approach was able to detect APOBEC3A/B induced mutations in next-generation sequencing data, despite background from polymerase errors, because the errors observed after amplifying the same sequence from a plasmid template only rarely resulted in cytosine deaminations at APOBEC3A/B target sites (Figure 1A). APOBEC3A/B mutations were detected in DNA amplified from kidney biopsies, urine, and plasma, with no clear quantitative differences between the three sample types. Urine samples, which are often easier to obtain because of their non-invasive nature, can therefore be used to study the impact of APOBEC3A/B on the BKPyV population in patients.

We found no difference in APOBEC3A/B mutation rates between BKPyV genotypes, but in PCR products amplified from urine samples, there was a clear association between APOBEC3A/B mutation rate and viral load. To some extent, this may be related to sampling—at low viral loads there may not be enough template molecules in the PCR reaction to efficiently detect mutations present at frequencies ~0.1%. This may explain why we did not detect any plasma or biopsy samples with >20 APOBEC3A/B mutations per 10^4^ read pairs, since viral loads in DNA extracted from these samples were lower than those in DNA extracted from urine. However, this potential bias cannot explain all of our observations, because high urine viral loads were not always accompanied by high APOBEC3A/B mutation rates. In some individuals, high APOBEC3A/B mutation rates lagged behind peak viral loads, while in others, APOBEC3A/Bmutation rates were consistently low, despite high viral loads. The first of these two observations is consistent with APOBEC3A induction by type-I interferons [35,36] subsequent to virus replication, while the second suggests that the activity of APOBEC3A/B editing is not the same in all individuals. This was illustrated particularly in patients 2017-20 and 2018-22, in whom the viral load profiles were highly similar, but while the APOBEC3A/B mutation rate tracked viral load in patient 2017-20, relatively few APOBEC3A/B mutations were detected in samples from patient 2018-22. These observations suggest that APOBEC3A/B editing rates vary between individuals in the context of BKPyV infection, and this may constitute a risk factor for the development of urothelial carcinoma subsequent to BKPyV infection, due to the role of APOBEC3A/B as a major mutagen in this cancer [37,38]. In terms of host genetics, there is a deletion/insertion polymorphism at the *APOBEC3* locus, in which the deletion allele results in loss of *APOBEC3B*. This allele is relatively rare in Western Europe, but presents at frequencies approaching 0.5 in East Asia, including Vietnam [39]. Interestingly, presence of the APOBEC3B deletion allele was found to be associated with higher levels of APOBEC3 DNA editing in oral squamous cell carcinoma samples [40], indicating that this genetic polymorphism does affect APOBEC3 mutation rates in vivo. Although our initial results did not reveal any major differences in the rate of APOBEC3A/B mutations in patients from Nantes and Hanoi, further work will be required to establish whether there is a link between kidney donor and/or receiver *APOBEC3B* deletion/insertion genotype, and the APOBEC3A/B mutation rates observed in BKPyV genomes.

Since PyVAN is associated with higher viral loads, the association between viral load and APOBEC3A/B mutation rate that we describe here is consistent with previous work [6] which documented mutations at APOBEC3A/B sites in NGS data from 3 KTx recipients with PyVAN, but did not find evidence of APOBEC3A/B editing in 23 patients with active BKPyV replication in the absence of PyVAN. However, it is important to note that although only low levels of APOBEC3A/B editing were detected in samples from patients without PyVAN, the values observed in some urine samples were still significantly higher than those observed in plasmid controls, suggesting that a low level of APOBEC3A/B mutagenesis may occur in the absence of PyVAN, perhaps via the induction of APOBEC3A and APOBEC3B in the urothelium [9]. The identification of significant differences in APOBEC3A/B mutation rates between individuals raises the question of how APOBEC3 editing influences the clinical course of PyVAN. High rates of APOBEC3 editing have been associated with resolution of HPV16 infection in low-grade cervical lesions [41], and if a similar antiviral effect also exists in the context of PyVAN, one might expect individuals with higher APOBEC3A/B mutation rates to eliminate the virus more effectively, although the results observed in patient 2018-28, who had persistent high-level virus replication over 3 years, together with consistently high APOBEC3A/B mutation rates, suggest that this is not the case. Alternatively, the BKPyV variants that persist in the face of high APOBEC mutation rates may be attenuated and display diminished pathogenic potential. On the other hand, higher APOBEC3A/B mutation rates may be required for the virus to evade host immune responses [6], and could therefore be associated with virus persistence, with or without chronic PyVAN. Furthermore, individuals with higher APOBEC3A/B mutation rates may have a higher risk of developing urothelial carcinoma subsequent to PyVAN, due to the mutagenic effect of APOBEC enzymes on the host genome. The ability to measure the rates of APOBEC3A/B mutagenesis in BKPyV genomes excreted in the urine should make it possible to study a large cohort of patients in order to address these questions. 

Mechanistically, the key molecular players that link high viral loads, PyVAN, and stronger APOBEC3A/B editing are the viral t-antigens. Detection of LTAg, which directly drives polyomavirus genome replication in tubular epithelial cells, is what defines PyVAN, and both the LTAg and stAg induce APOBEC3B expression via the LXCXE motif [42]. The role played in this process by rearrangement of the BKPyV NCCR region, which leads to increased expression of early transcripts [43], remains to be elucidated. The principal function of the LXCXE motif is to drive entry into the cell cycle by binding to the tumor suppressor Rb and liberating E2F transcription factors from Rb/E2F complexes. Since cell cycle entry is an essential step for small DNA viruses that depend on multiple host co-factors for viral DNA replication, LXCXE motifs with similar functions are also found in the HPV E7 protein and the adenovirus E1a protein. Transcriptional induction of APOBEC3B has recently been described during adenovirus infection [44], although the molecular mechanism involved has not yet been identified, while in HPV infection, induction of APOBEC3B expression is dependent on the E6 protein [45,46], which does not contain an LXCXE motif, rather than the E7 protein. Overall, the available data indicate that although dissociation of Rb/E2F complexes by early proteins carrying the LXCXE motif is an important mechanism involved in APOBEC3B induction during viral infection, additional signals are likely to be required for high level APOBEC3B expression. 

In addition to SBS2 and SBS13, corresponding to APOBEC3A/B editing, the mutational signature SBS89 was consistently detected in clinical samples, and was also detected in viruses replicating in culture, suggesting that SBS89 represents mutations occurring during BKPyV replication. Unlike APOBEC3A/B mutations, the SBS89 mutation rate was not significantly correlated with viral load, and did not show detectable variation between individuals, and these observations are both consistent with a process that occurs at a constant rate during virus replication. The SBS89 mutational profile was described in normal colonic epithelial cells [47], its etiology is unknown, and it has not previously been linked to virus infection. In particular, in a study of HPV16 whole genome sequences from more than 5000 patients, Zhu et al. [41] identified the mutational signatures SBS2 (APOBEC3), SBS32, SBS26, and SBS9, but not SBS89. Although mutagenic processes acting on HPV are not necessarily the same as those acting on polyomaviruses, this raises the possibility that we may have mis-identified the SBS89 signature. Indeed, our study has two important methodological limitations that compromise the extraction and fitting of SBS signatures. 

Firstly, analyzing mutations at frequencies lower than 1% in NGS data raises the question of how to discriminate real mutations present in the virus population from polymerase errors introduced during PCR. To do this, we amplified multiple control amplicons from plasmids, and used the mutations observed in this data to exclude certain types of mutations from our analysis. Mutations in these control samples were fitted to the signatures SBS9, SBS38, SBS14 and, to a lesser extent, SBS10a, SBS20, and SBS4, making it difficult or impossible to detect authentic signals in the clinical samples corresponding to these SBS profiles. This was a particular problem with the SBS4 signal, because unlike SBS89, which showed the same tendency in all types of clinical sample, an increased SBS4 mutation rate compared to controls was only observed in kidney biopsies (Figure 1B,C). This suggests two possible interpretations—either the increased SBS4 mutation rate in biopsies may be a statistical artifact, related to the limited number of biopsy samples analyzed, or it may be a real signal that we could not detect reliably because polymerase errors amplified during PCR also generated an SBS4 signature. At the present time, our data do not allow us to distinguish between these two possibilities. SBS4 is the signature of mutations induced by tobacco smoking [48], so it is plausible that KTx recipients with BKPyV reactivation who smoke could have high rates of SBS4 mutations in BKPyV sequences. Exploration of this hypothesis will require more accurate identification of SBS4 mutations than we achieved in this report.

Secondly, sequence bias in the short *VP1* region that we studied could lead to mis-attribution of SBS signatures. Since genotype differences are concentrated in the *VP1* typing region, this may be related to the observation that SBS89 mutations were more frequently detected in genotype II and genotype IV viruses. In future studies it will be important to correct these two methodological biases by studying whole genome sequences amplified by a DNA polymerase with higher fidelity than that used here. 

## Figures and Tables

**Table 1:** Barcoded primers for VP1 amplification.

Forward Primer	Sequence	Reverse Primer	Sequence
VPS1-1	TGATCACGCAAGTGCCAAAACTACTAAT	VPS2-1	CAATCACGTGCATGAAGGTTAAGCATGC
VPS1-2	TGCGATGTCAAGTGCCAAAACTACTAAT	VPS2-2	CACGATGTTGCATGAAGGTTAAGCATGC
VPS1-3	TGTTAGGCCAAGTGCCAAAACTACTAAT	VPS2-3	CATTAGGCTGCATGAAGGTTAAGCATGC
VPS1-4	TGTGACCACAAGTGCCAAAACTACTAAT	VPS2-4	CATGACCATGCATGAAGGTTAAGCATGC
VPS1-5	TGACATGTCAAGTGCCAAAACTACTAAT	VPS2-5	CAACATGTTGCATGAAGGTTAAGCATGC
VPS1-6	TGGCCAATCAAGTGCCAAAACTACTAAT	VPS2-6	CAGCCAATTGCATGAAGGTTAAGCATGC
VPS1-7	TGCAGATCCAAGTGCCAAAACTACTAAT	VPS2-7	CACAGATCTGCATGAAGGTTAAGCATGC
VPS1-8	TGGATCAGCAAGTGCCAAAACTACTAAT	VPS2-8	CAGATCAGTGCATGAAGGTTAAGCATGC

## Data Availability

The data presented in this study, notably Illumina sequence reads, are available on request from the corresponding author. The data are not publicly available due to the limited benefit of maintaining several hundred thousand reads of the same BKPyV genome region, relative to the environmental cost of keeping this data available on servers. Intermediate data, such as .vcf files, and the BKPyV BSGenome object are also available on request.

## Supplementary Materials

The following supporting information can be downloaded at: https://www.mdpi.com/article/10.3390/v14092077/s1, Table S1: Reference BK genome sequences used for variant calling.
