# Supplementary material for: Quantification of APOBEC3 Mutation Rates Affecting the VP1 Gene of BK Polyomavirus In Vivo
Source: Viruses. 2022 Sep 19;14(9):2077. doi: 10.3390/v14092077 (PMC9504301; doi:10.3390/v14092077)
Supplement: Supplementary file 1 [file viruses-14-02077-s001.zip › viruses-1886718-supplementary table.pdf]

Supplementary Table

Table S1. - reference BK genome sequences used for variant calling.

Genotype	Sequence name / isolate	Genbank accession
gIa	MM	V01109
gIb2*	PittVR2*	DQ989796
gII	GBR-12	AB263920
gIII	KOM-3	AB211386
gIVc2	A-66H	AB369093

* For variant calling, the PittVR2 sequence was manually edited to revert two VP1 mutations (73K and 82D) present in that isolate to their wild-type equivalents (73E and 82E).
